# Allyl isothiocyanate depletes glutathione and upregulates expression of glutathione S-transferases in *Arabidopsis thaliana*

**DOI:** 10.3389/fpls.2015.00277

**Published:** 2015-04-22

**Authors:** Anders Øverby, Ragni A. Stokland, Signe E. Åsberg, Bjørnar Sporsheim, Atle M. Bones

**Affiliations:** Department of Biology, The Norwegian University of Science and TechnologyTrondheim, Norway

**Keywords:** allyl isothiocyanate, isothiocyanates, Arabidopsis, glutathione, plant defense, glutathione transferase

## Abstract

Allyl isothiocyanate (AITC) is a phytochemical associated with plant defense in plants from the Brassicaceae family. AITC has long been recognized as a countermeasure against external threats, but recent reports suggest that AITC is also involved in the onset of defense-related mechanisms such as the regulation of stomatal aperture. However, the underlying cellular modes of action in plants remain scarcely investigated. Here we report evidence of an AITC-induced depletion of glutathione (GSH) and the effect on gene expression of the detoxification enzyme family glutathione S-transferases (GSTs) in *Arabidopsis thaliana*. Treatment of *A. thaliana* wild-type with AITC resulted in a time- and dose-dependent depletion of cellular GSH. AITC-exposure of mutant lines *vtc1* and *pad2-1* with elevated and reduced GSH-levels, displayed enhanced and decreased AITC-tolerance, respectively. AITC-exposure also led to increased ROS-levels in the roots and loss of chlorophyll which are symptoms of oxidative stress. Following exposure to AITC, we found that GSH rapidly recovered to the same level as in the control plant, suggesting an effective route for replenishment of GSH or a rapid detoxification of AITC. Transcriptional analysis of genes encoding GSTs showed an upregulation in response to AITC. These findings demonstrate cellular effects by AITC involving a reversible depletion of the GSH-pool, induced oxidative stress, and elevated expression of GST-encoding genes.

## Introduction

**Figure 1:**
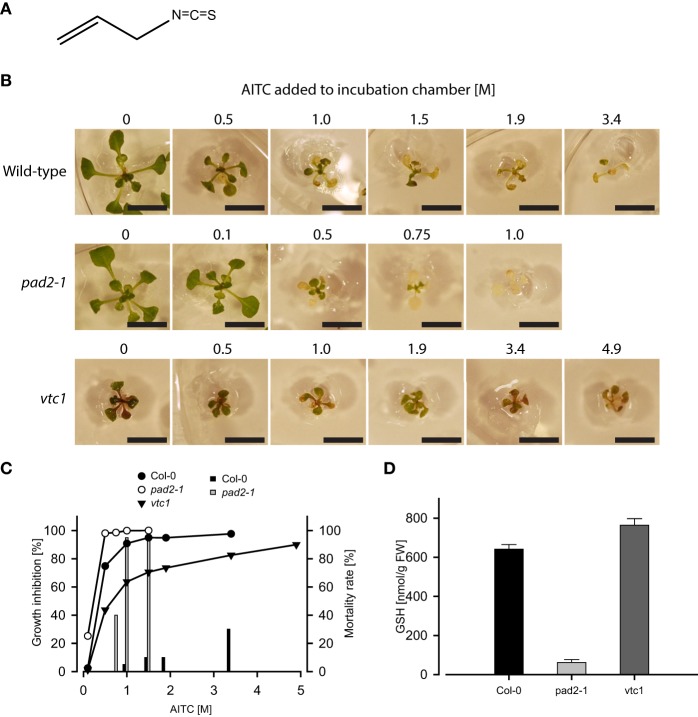
**Exposure to vapor of AITC (A) for 1 h inhibits growth of *A. thaliana* wild-type and mutant lines *pad2-1* and *vtc1* (B)**. Bars represent 1 cm. These lines respond differently in both growth inhibition and mortality following AITC-exposures **(C)**. Graphs depict growth inhibition of seedlings of wild-type (11 d), *pad2-1* (11 d), and *vtc1* (13 d) plants relative to mock control. Bars represent mortality rates. **(D)** GSH content estimated in Col-0 and the mutant lines *pad2-1* and *vtc1* showed decreased and enhanced GSH-levels, respectively. Values are mean ± std. dev. of 3 replicates.

Isothiocyanates (ITCs) are biodegradation products found in several plants belonging to the Brassicaceae family, and are produced upon disruption of the tissue by herbivores or pathogens. ITCs are stored as their biologically inert precursors glucosinolates that is physically separated from the hydrolyzing enzyme myrosinase. Upon damage to the cells both substrate and enzyme is released and the subsequent hydrolysis of glucosinolate yields several products including ITCs (Halkier and Gershenzon, 2006; Kissen and Bones, 2009). Being volatile and exerting a strong and pungent taste, ITCs are meant to repel herbivores (Lambrix et al., 2001; Yan and Chen, 2007). In addition, ITCs are highly reactive, making them an efficient countermeasure against pathogens at the site of the wound (Olivier et al., 1999). ITCs are composed of an –N=C=S reactive group linked to an R moiety dictating other physiochemical properties. Previously studied examples include the aliphatic allyl ITC (AITC; see Figure 1A for chemical structure) and sulforaphane (SFN), and the aromatic benzyl ITC (BITC), and phenethyl ITC (PEITC). Members of the Brassicaceae family include several vegetables commonly consumed by humans such as broccoli, brussels sprouts, watercress, wasabi, cauliflower and mustard. ITCs are most commonly known for their cancer preventive effects shown through epidemiological studies that reported a reduced risk of certain cancer types when consuming these vegetables (Seow et al., 2002; Brennan et al., 2005; Tang et al., 2008). These discoveries have led to numerous studies aiming to elucidate the molecular mechanisms underlying the cancerpreventive properties of ITCs (Navarro et al., 2011; Zhang, 2012). Although the underlying mechanisms are not fully understood, it is well known that several mechanisms govern the activity of ITCs in cancerous cells (Navarro et al., 2011). ITCs are able to antagonize multiple targets due to an electrophilic central C-atom in the reactive part, which can conjugate with any accessible thiol group (Figure 1A). In addition to cysteine containing proteins, the abundantly present GSH presents an inevitable target for ITCs as demonstrated with AITC (Kawakishi and Kaneko, 1985; Zhang et al., 1995). Activity of ITCs in plants has also been reported. Previous reports of ITCs or ITC-deriving extracts in weed control have shown promising results (Boydston and Hang, 1995; AlKhatib et al., 1997; Haramoto and Gallandt, 2005; Norsworthy et al., 2006). ITC-induced growth inhibition has been reported in wheat, velvet leaf, palmer amaranth, lettuce and the model plant *Arabidopsis thaliana* (Wolf et al., 1984; Bialy et al., 1990; Yamane et al., 1992; Norsworthy and Meehan, 2005; Hara et al., 2010). More recently, a physiological role for ITCs in defense has been proposed. Khokon and colleagues showed that exposure of *A. thaliana* to AITC led to a stomatal closure, possibly to prevent water loss and intrusion of microorganisms trough stomata (Khokon et al., 2011). Moreover, a role in protection against heat stress has also been reported (Hara et al., 2013). To fully understand the presumable physiological purpose of ITCs, more knowledge about the molecular events initiated in plant cells by ITCs is required. We found previously that exposure of *A. thaliana* to ITCs results in disintegration of microtubules and upregulation of stress-related genes including *HSP70, HSP90, DNAJ*, and *STZ* (Øverby et al., 2015). Furthermore, we found that exposure of *A. thaliana* to AITC results in reduction of actin-mediated intracellular transport (Sporsheim et al., submitted). Moreover, AITC was able to induce a shift in the cell cycle distribution in *A. thaliana* (Åsberg et al., submitted). The metabolite GSH functions in metabolism, mediating redox signaling, and detoxification of xenobiotics (reviewed in Noctor et al., 2012). The detoxification of ITCs by GSH is governed by glutathione S-transferases (GSTs) that catalyze the conjugation of thiol group from GSH with the electrophilic central C-atom in ITCs. *A. thaliana* possesses 55 GSTs categorized into 7 classes, and they constitute a robust machinery with broad target specificity and to a certain extent overlapping functions (Dixon et al., 2010). However, the response of these enzymes upon ITC-exposure is yet unknown. In the present report we employed the aliphatic and highly volatile AITC to study the effect of AITC on the GSH-pool as well as the transcriptional response of genes encoding GSTs in *A. thaliana*. The potential role of AITC-induced change in the GSH-pool in connection with a physiological response in *A. thaliana* is discussed. This study contributes to an increased understanding of the cellular effects of ITCs in plants and shed light on the role of AITC *in vivo*.

## Methods

### Chemicals

AITC (95% purity), Murashige-Skoog (MS), kit for determination of total glutathione content, 2′,7′-dichlorodihydrofluorescein diacetate (H_2_DCFDA), Spectrum Plant Total RNA Kit and monodansylcadaverine (MDC) were purchased from Sigma Aldrich (Norway). Primers for qPCR were synthesized by Eurofins MWG (Germany). QuantiTect Reverse Transcription Kit and RNase-Free DNase Set were purchased from QIAGEN (Norway). SYBRgreen was purchased from Roche Applied Science (Norway).

### Plant growth and AITC-treatment

*A. thaliana* wild-type was ecotype Col-0. Mutant lines *pad2-1* and *vtc1* were also employed. These lines possess reduced and increased levels of GSH, respectively (Pavet et al., 2005; Parisy et al., 2007). For autophagy studies, a transgenic line expressing GFP-tagged ATG8a was employed (Svenning et al., 2011). Before sowing, seeds were subjected to a chlorine/ethanol-based disinfection procedure. Disinfected seeds mixed with agarose (0.1 % w/v) were sowed onto solid Murashige-Skoog (2.15 g/l MS, 20 g/l sucrose and 6 g/l agar consisting of agar from Bacto and Phyto mixed 1:1) medium in a 9 cm petri-dish. The plate was incubated for 2 days at 4°C in the absence of light for vernilisation of seeds. The plate was then incubated in a 16/8 h day/night cycle at room temperature allowing the plants to grow. For ITC-treatment, a 9 cm petri-dish containing 11-days old wild-type or *pad2-1*, or 13-days old *vtc1* (due to the reduced growth rate of this mutant line, additional time was given before treatment in order to reach a comparable stage to the wild-type and *pad2-1* mutant line) on MS-agar without lid was placed in a 13 cm petri-dish together with a filter paper added 200 μl of an AITC-dilution. The latter mentioned plate was sealed and incubated in room temperature for the desired time of AITC-exposure before transferring 10 plants to a 9 cm petri-dish containing fresh MS-agar medium with a slot for each plant subsequently filled with agarose to cover roots and avoid drought stress. Plants were then incubated as above and growth was monitored by capturing pictures every day for 9 days after treatment. The software ImageJ was used to estimate the diameter of the plants.

### Glutathione assays

Roots from untreated or AITC-treated seedlings were removed before snap-freezing in liquid nitrogen followed by grounding of plant material using a TissueLyser. 5-sulfosalicylic acid (5% w/v; 500 μl/100 mg fresh weight) was added to the grounded tissue for extraction and deproteinization. The supernatant was purified by centrifugation before assayed for GSH-content. The level of total glutathione was measured using a glutathione assay kit according to the provided protocol.

### Chlorophyll determination

Chlorophyll content was determined as described previously with minor modifications (Gao and Zhang, 2008). Briefly, approximately 100 mg of leaves from *A. thaliana* plants were grounded in liquid nitrogen followed by extraction using 80% acetone and centrifugation. The collected supernatants were analyzed spectrophotometrically at wavelengths 645 and 663 nm to determine chlorophyll a and b contents, respectively, (Arnon, 1949).

### ROS determination

Following treatment with AITC, wild-type seedling of *A. thaliana* was submerged and incubated in a reaction buffer containing 50 μM H_2_DCFDA (2′,7′-dichlorofluorescin diacetate), 5 mM KOH, 50 μM CaCl_2_, 10 mM MES-Tris and pH 6.15 for 30 min, followed by washing in the same buffer without H_2_DCFDA. Pictures were captured through a fluorescence microscope.

### qPCR of AITC-treated *A. thaliana*

Quantitative PCR was performed as previously described (Øverby et al., 2015). Briefly, fresh plant material was snap-freezed in liquid nitrogen and subsequently grounded from which total RNA was extracted using Spectrum Plant Total RNA Kit. RNase-Free DNase Set was used to prevent DNA contamination. RNA concentration was measured using NanoDrop 1000 (Thermo Scientific). cDNA was synthesized using QuantiTect Reverse Transcription Kit, and qPCR was performed with SYBRgreen in 96-well plate in a Lightcycler 480 (Roche Applied Science) with the following program: preincubation step (95°C, 5 min), 45 amplification cycles (95°C, 10 s; 55°C, 10 s; 72°C, 10 s), and a final melting curve analysis. Cycle threshold values, PCR efficiencies and relative expression values were calculated using Lightcycler 480 Software (Roche), LinRegPCR and REST 2009 (QIAGEN), respectively. At4g24550 (*Clathrin*) and At4g34270 (*TIP41-like*) were used as housekeeping genes. Primer sequences are given in Supplementary Table S1.

### MDC-staining

Staining of autophagosomes with MDC followed the protocol as previsously published (Contento et al., 2005). Briefly, treated or untreated seedlings of *A. thaliana* were stained with 0.05 mM MDC dissolved in phosphate buffered saline (PBS) and subsequently washed three times in PBS. Detection was visualized by fluorescence microscopy.

## Results and discussion

### AITC-induced growth inhibition of *A. thaliana* is influenced by the intracellular GSH-pool

Exposure of 11-days old *A. thaliana* wild-type seedlings to vapor-phase of AITC in the concentration range 0.5–3.4 M for 1 h displayed a dose-dependent growth inhibition consistent with results from a parallel study (Øverby et al., 2015; Figure 1B). Although a different exposure method was applied, Hara and colleagues also showed AITC to exert an effect on *A. thaliana* growth (Hara et al., 2010). The spraying method employed by Hara et al. was also attempted in our studies, but reproducible data were failed to achieve possibly due to an uneven distribution and the instability of AITC in a liquid solution (data not shown) (Ohta et al., 1995). We therefore continued treatment by exploiting the volatility of AITC in our experiments. In addition to growth retardation, AITC treatment caused bleaching of leaves at 0.5 M, which was increased by exposure to higher concentrations (Figure 1B), an effect previously reported by Hara et al. showing that bleaching was also induced by methyl ITC (MITC) and phenethyl ITC (PEITC) (Hara et al., 2010). ITCs bind readily to thiol-containing GSH to form conjugates and disturbs the intracellular redox balance (Kawakishi and Kaneko, 1985). The resulting generation of reactive oxygen species (ROS) following conjugation to and depletion of GSH has also been demonstrated in mammalian cells (Zhang, 2000; Singh et al., 2005; Xiao et al., 2006). To investigate the significance of the intracellular GSH-pool upon AITC-exposure in *A. thaliana*, we employed the mutant lines *pad2-1* and *vtc1*, which possess reduced and increased GSH-levels, respectively, (Pavet et al., 2005; Parisy et al., 2007). Quantification of total GSH content in these lines revealed that the *pad2-1* seedlings contained 10% and the *vtc1* seedlings contained 119% GSH of that in wild-type (Figure 1D). Seedlings of *pad2-1* proved to be more susceptible to AITC-induced growth inhibition compared to wild-type seedlings of the same age (Figures 1B,C). Wild-type seedlings exposed to vapor of 0.1 M AITC for 1 h did not display any aberration in growth or bleaching. In contrast, *pad2-1* seedlings showed significant growth reduction (>20%) and displayed some bleaching of the leaves. Moreover, *pad2-1* seedlings showed increased mortality when exposed to AITC. Vapor of 1 M AITC for 1 h caused 95% lethality whereas only 5% of the wild-type seedlings died from the same treatment (Figure 1C). The knock-out line *vtc1* possesses reduced ascorbic acid content, displays microlesions and a reduction in growth when compared to Col-0 seedlings of the same age (Pavet et al., 2005). However, upon AITC-treatment the relative growth inhibition of *vtc1* was reduced compared to that of wild-type (Figures 1B,C). Interestingly, none of the AITC doses used in this study proved to be lethal to the *vtc1* seedlings. Although future studies on the physiological effects of ITCs should include other lines with aberrated levels of GSH, the present study indicates that the inhibitory effects of AITC on the growth of *A. thaliana* may be linked to the intracellular GSH pool.

### AITC causes depletion of GSH in *A. thaliana*

**Figure 2:**
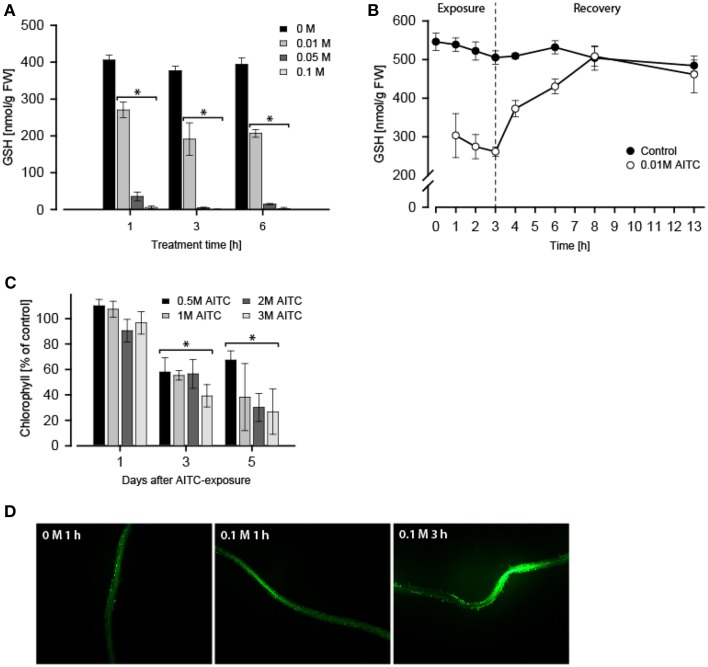
***A. thaliana* seedlings (11 d) exposed to 0.01–0.1 M AITC for 1–6 h displayed a dose- and time-dependent depletion of GSH-pool. (A)** An effect which proved to be reversible when AITC-treated seedlings (0.01 M) were allowed to recover in an AITC-free environment **(B)**. Values represent means ± s.d. of 3 replicates. The stapled line depicts the time when AITC-treated seedlings were transferred for recovery. *A. thaliana* (11 d) also displayed loss of chlorophyll 1–5 days following 1 h 0.5–3 M AITC-treatments **(C)**. Values represent means ± s.d. of 4 replicates. **(D)** Staining of ROS in roots with H_2_DCFDA following treatment with vapor of AITC showed an increased intensity when observed under a fluorescene microscope. Statistical analysis was performed with a two-tailed *t*-test, ^*^p < 0.05.

Next, we investigated the effect of AITC-exposure on the GSH-pool in *A. thaliana*. Treatment of 11-days old *A. thaliana* wild-type with vapor of 0.1 M AITC for 1 h did not cause growth inhibition (Figures 1B,C), but measurement of free GSH-level following this treatment revealed an almost complete depletion of GSH (Figure 2A). Treatments with 0.05 and 0.01 M AITC resulted in reduction to 9 and 67% of the GSH estimated in control plants, respectively. Increased incubation time with 0.01 M AITC for 3 h further depleted the GSH-pool to 51% of that in controls (Figure 2A). Interestingly, increasing the incubation time to 6 h did not yield any further significantly change in GSH-level or the relative reduction of GSH (Figure 2A), suggesting a mean to sustain or regain the GSH-pool imbalanced by AITC. This was further explored in a time-series experiment in which the GSH-content was measured in mock- or 0.01 M AITC-treated plants for up to 3 h followed by a recovery period in which plants were incubated in an AITC-free atmosphere (Figure 2B). Following 3 h incubation and an initial reduction in the GSH pool, a rapid recovery of GSH was observed. After 5 h recovery the plants regained the same GSH-level as maintained in control plants. These findings suggest that AITC depleted the intracellular GSH-pool in *A. thaliana* and that the depletion may be efficiently reversed. In addition, a preliminary test was performed to investigate the effect on GSH/GSSG-balance, showing a reduction by AITC in accordance with findings from mammalian cell types (data not shown) (Xu and Thornalley, 2001; Tusskorn et al., 2013). As mentioned above, AITC induces oxidative stress upon entering a cell. In *A. thaliana*, this was observed by monitoring chlorophyll content and ROS level in the roots subsequent to AITC exposure (Figures 2C,D). Two days after treatment with vapor of 0.5, 1, 2, and 3 M AITC for 1 h a loss of chlorofyll was detectable as evident by bleaching of the leaves. Three days after exposure, the chlorofyll content was approximately halved for all doses tested with a further minor decrease after 5 days (Figure 2C). These results are consistent with a previous report showing AITC as well as MITC and PEITC to induce bleaching and loss of chlorophyll in *A. thaliana* (Hara et al., 2010). Inspection of ROS after staining with H_2_DCFA showed that vapor of AITC increased the ROS-content in roots (Figure 2D). Results from seedlings subjected to 0.1 M AITC for 1 and 3 h are shown, but similar results were found when applying 0.05 M for 3 h and 2 and 3 M for 1 h (data not shown). However, local variations in the roots complicated quantification of ROS-content and the impact of different doses could therefore not be distinguished. Experiments revolving the effect of AITC on mitochondria in transgenic *A. thaliana* expressing YFP-tagged mitochondria were also undertaken. Preliminary results indicated a morphological change of the mitochondria (data not shown). In addition, the movement of the mitochondria was stagnated following AITC-treatment, which coincides with the results from a parallel report in which the treatment of *A. thaliana* with AITC resulted in inhibition of actin-mediated intracellular transport (Sporsheim et al., submitted). ITC conjugating with GSH affecting the mitochondria has also been reported in mammalian cells, usually resulting in damage and reduced functionality of mitochondria (Nakamura et al., 2002; Zhang et al., 2003, 2005). Autophagy is an intracellular degradation process involved in removing damaged proteins and organelles, and can be induced by a number of stressors including oxidative stress (Bassham, 2007). We therefore hypothesized that AITC induced oxidative stress could induce autophagy in *A. thaliana*. Expression of several autophagy-related genes including *ATG8H, ATG18B*, and *ATG18C* were upregulated by subjection to vapor of 0.02–0.1 M AITC. However, when detection methods such as MDC staining of autophagic vesicles and a transgenic line expressing the autophagy-associated protein ATG8e tagged with GFP were employed, a reproducible response in autophagy subsequently to treatment was not obtained (data not shown). This may be linked to the difficulties with these methods as previously described (Mitou et al., 2009). BITC has previously been shown to induce autophagy through inhibition of mTOR in human prostate cancer cells (Lin et al., 2013). A similar response in plant cells might be hypothesized and future work should aim at elucidating the effect of ITCs on the autophagic machinery in plants. Although it is established that AITC conjugates with GSH causing oxidative stress, whether or not this reaction is linked to a physiological response remains unclear. GSH is a metabolite with various functions in plant cells including roles in detoxification of xenobiotics, modulation of redox balance and plant growth and development (reviewed in Noctor et al., 2012). GSH has previously also been linked to the regulation of stomatal closure in *A. thaliana* (Akter et al., 2012). Briefly, closure of stomata operates via abscisic acid (ABA) and methyl jasmonate (MeJA) to elevate ROS-levels that subsequently results in a stomatal closure. A previous study showed that exposure of *A. thaliana* to AITC resulted in a dose-dependent closing of stomata accompanied by elevated levels of ROS and nitric oxide (NO) (Khokon et al., 2011). Interestingly, the same study showed that externally supplied GSH blocked the AITC-induced closing of stomata (Khokon et al., 2011). MeJA is a key element in the regulation of stomatal opening and closure and may increase ROS levels to promote stomatal closure also when GSH levels are high. This suggests that GSH may exert an inducer/inhibitor effect on this pathway rather than being an essential signaling component (Akter et al., 2010, 2013). This is further supported by the findings that externally supplied GSH blocks stomatal closing (Khokon et al., 2011; Akter et al., 2013), and that GSH depletion enhances MeJA-induced stomatal closure (Akter et al., 2013). We showed that AITC induces a depletion of GSH and an increase in ROS in the roots of *A. thaliana* (Figures 2A,D). Previous studies showed AITC exposure of *A. thaliana* to coincide with increased ROS in guard cells (Hossain et al., 2013). Interestingly, the ABA/MeJA-mediated increase in ROS and stomatal closure was inhibited in the tgg1-3 and tgg2-1 double mutant but not in any of the single mutant lines (Islam et al., 2009). Despite the unclear role of AITC in regulation of stomatal closure, this may suggests that the hydrolysis products of glucosinolates could be involved in controlling stomatal aperture. A possible mechanism may involve an inhibitory effect of GSH that suppresses the MeJA/ABA-induced stomatal closure which in turn is relieved when a GSH-depleting agent (e.g., AITC) interferes. We also found that following AITC-induced GSH depletion, a rapid repletion is obtained if plants were allowed to recover in an AITC-free environment (Figure 2B). This is a scenario likely to mimic the effect of AITC in nature as this compound would be released in pulses rather than a steady dose for a long time upon tissue damage, allowing the cells to recover following an exposure. As prolonged high levels of ROS could be damaging to the cell components, the repletion of GSH could be a means to neutralize AITC-induced ROS and thus protect the cell internal components. GSH level has also been linked to pathogen resistance in plants. The mutant lines *pad2, cad2*, and *rax1-1* have reduced level of GSH and are more susceptible to some pathogens (Ball et al., 2004; Schlaeppi et al., 2008). It has been suggested that a sufficient GSH pool is required in later responses to pathogens (Noctor et al., 2012). This would imply that GSH could serve different roles during the course of tissue damage and potential intrusion by microorganisms. At early stages, GSH-related regulation of MeJA/ABA-induced stomatal closure might serve to protect the leaf from attacking pathogens. At later stages, GSH might have a role in defensive hormone and secondary metabolite production. In a parallel study, we showed that long-term exposures (36, 48, and 60 h) of *A. thaliana* to AITC affected the distribution of cells in the cell cycle leading to an accumulation of cells in the S-phase (Åsberg et al., submitted). Little is known of the role of GSH in the plant cell cycle. Interestingly, nuclear recruitment of GSH during the G_1_- and S-phase of the cell cycle has been shown to be important for the redox state of the cytoplasm and redox-related genes (Pellny et al., 2009; Diaz Vivancos et al., 2010a,b). Although GSH-levels were not measured in our studies revolving the effect of AITC on cell cycle in *A. thaliana*, future studies should be undertaken to investigate the role of ITCs in nuclear translocation of GSH.

### AITC induces upregulation of GST-encoding genes

**Figure 3:**
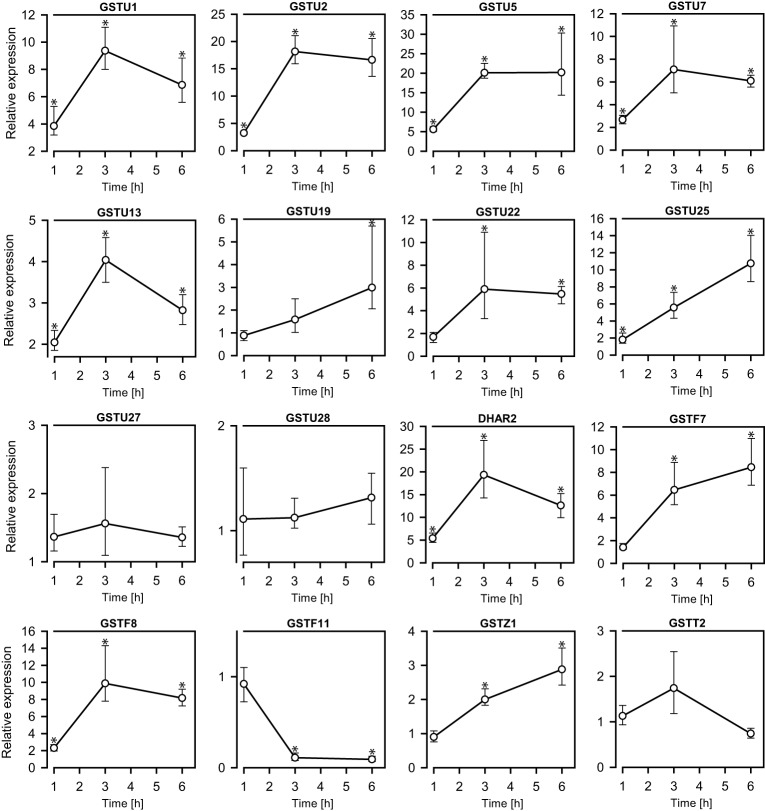
**The exposure of 0.05 M AITC to *A. thaliana*seedlings (11 d) for 1–6 h led to changes in the expressions of genes encoding GSTs**. The relative expression levels were determined by qPCR against a mock-treated control. Stastitical calculations were performed using the software REST 2009. Values represent means ± s.d. of 3–4 replicates. ^*^p < 0.05.

GSTs comprise a family of enzymes commonly associated with detoxification and upregulated activity in response to various types of stress. Transcriptional analysis of a set of GST-encoding genes was therefore undertaken in order to investigate the response to AITC-exposure. Among the 55 GSTs in *A. thaliana*, 18 were analyzed for regulation in response to AITC-exposure representing 5 of the 7 classes into which the GSTs are categorized (Table 1); *GSTU1, GSTU2, GSTU3, GSTU5, GSTU7, GSTU13, GSTU19, GSTU22, GSTU25, GSTU27, GSTU28, DHAR2, GSTF6, GSTF7, GSTF8, GSTF11, GSTZ1*, and *GSTT2*. Exposure to 0.05 M AITC for 1 h resulted in upregulation of 10 of these genes (Figure 3, Table 1). After 3 h an additional 3 genes were upregulated and 1 was downregulated. Following 6 h of exposure, 1 additional gene was upregulated. Time-dependency was most distinct between 1 and 3 h treatments where most of the genes showed an increased expression (Figure 3). A dose-dependent effect was also observed when plants were treated with 0.1 M AITC for 1 h, resulting in the upregulation of 8 genes and downregulation of 1 gene (Table 1). The genes *GSTU3* and *GSTF6* in control plants appeared to be expressed at a level outside the detection limit for our qPCR analysis method. However, after treatments with AITC these genes were expressed within the detection limit. They were there therefore interpreted as upregulated despite that an estimate of the relative expression could not be performed. In a parallell study by our group, we tested the effect of 0.5 M AITC on the expression of the genes *GSTF6, GSTF7*, and *GSTU19* (which were found to be upregulated in the present study) without any significant response (Øverby et al., 2015). These data together with a previous report suggests that a lower dose of AITC is more effective in promoting an enhanced gene expression of GSTs in *A. thaliana* (Hara et al., 2010). Furthermore a previous report showed that low doses of PEITC upregulate *GSTU19* and showed tendencies to upregulation of *GSTF6, GSTF7*, and *GSTZ1* (Hara et al., 2010). These were also found to be upregulated in our studies (Table 1), but the kinetics and degree of response were different. Moreover, *GSTU5, GSTU13*, and *GSTF8* that were upregulated by AITC in our studies, showed no response to PEITC (Hara et al., 2010). These findings indicate that ITC induced changes in expression of genes encoding GSTs may vary among different ITCs, although the effect of different exposure methods cannot be ruled out. Based on our findings, a significant number of genes encoding GSTs were upregulated in response to AITC, with differences in both the relative expression and the development of expression over time. GSTs comprise a class of enzymes with redundant functionality, and expression of several GSTs may be induced by different stimuli (Dixon and Edwards, 2010). Among the genes shown to be upregulated in the present study, 9 were found to be regulated by other stimuli including salicylic acid, H_2_O_2_, hypoxia, chilling and bacterial- and fungal stress in a previous report (Sappl et al., 2009). This may suggest that the response of the selected genes could reflect a general stress response rather than an ITC-specific response. GSH is synthesized in a two-step mechanism by the enzymes γ-EC synthetase and GSH synthetase transcribed from the genes *GSH1* and *GSH2*, respectively. A previous study with depletion of GSH in *A. thaliana* by cadmium and copper resulted in an upregulation of *GSH1* and *GSH2* (Xiang and Oliver, 1998). Interestingly, exposure of 0.05 M AITC for 1–6 h to *A. thaliana* did not result in an upregulation of *GSH1*, but upregulation of *GSH2* after 1 h of exposure. Our studies show genes of several GSTs to be upregulated in response to AITC, although further studies should be undertaken to uncover the role of GSTs in AITC-induced defense mechanisms in *A. thaliana*.

**Table 1:** **Transcriptional response of GST-encoding genes in *A. thaliana* based on qPCR analysis and stastitical analysis using the software REST 2009**.

		**0.05 M AITC**			**0.1 M AITC**
**Gene**	**Accession number**	**1 h**	**3 h**	**6 h**	**1 h**
*GSTU1*	At2g29490	+	+	+	+
*GSTU2*	At2g29480	+	+	+	+
GSTU3^*^	At2g29470	+	+	+	
*GSTU5*	At2g29450	+	+	+	+
*GSTU7*	At2g29420	+	+	+	+
*GSTU13*	At1g27130	+	+	+	+
*GSTU19*	At1g78380			+	−
*GSTU22*	At1g78340		+	+	+
*GSTU25*	At1g17180	+	+	+	
*GSTU27*	At3g43800				
*GSTU28*	At1g53680				
*DHAR2*	At1g75270	+	+	+	+
GSTF6^*^	At1g02930	+	+	+	
*GSTF7*	At1g02920		+	+	
*GSTF8*	At2g47730	+	+	+	+
*GSTF11*	At3g03190		−	−	
*GSTZ1*	At2g02390		+	+	
*GSTT2*	At5g41240				

*“+” depicts a significant upregulation (>1.6 relative expression) and “−” depicts a significant downregulation (<0.7 relative expression) when compared to mock-treated control*.

* *Expression levels low, see text*.

## Conclusions

In conclusion, the present study shows that AITC causes growth retardation, bleaching and induction of oxidative stress in *A. thaliana* seedlings. AITC was also shown to cause GSH depletion, followed by repletion and upregulation of *GST* encoding genes. Although more work is required to fully understand a presumable physiological role of AITC in plants, this study provides evidence of cellular effects induced by AITC in *A. thaliana*.

### Conflict of interest statement

The authors declare that the research was conducted in the absence of any commercial or financial relationships that could be construed as a potential conflict of interest.
