# Supplementary material for: Allyl isothiocyanate depletes glutathione and upregulates expression of glutathione S-transferases in Arabidopsis thaliana
Source: Front Plant Sci. 2015 Apr 22;6:277. doi: 10.3389/fpls.2015.00277 (PMC4406002; doi:10.3389/fpls.2015.00277)
Supplement: Supplementary file 1 [file Table1.DOC]

SUPPLEMENTARY TABLE S1. Primers used in the qPCR analyses with their sequences presented in 5’-3’ direction.

| Gene | Forward primer sequence 5’-3’ | Reverse primer sequence 5’-3’ |
| --- | --- | --- |
| *GSTU1* (At2g29490) | GATCAGACGTGGAAGAACAG | AAGGGACCTAAACCCTAGTG |
| *GSTU2* (At2g29480) | GGGCCAAGTTCGTTGATGAG | CATTCCCAAGCCCGAGCAAG |
| *GSTU3* (At2g29470) | AAGCCCATTCAGTCGTAGAG | TGACCTGAGCCCTATCATTG |
| *GSTU5* (At2g29450) | TTAGCCGTCGGGTCGAGATG | TCCTTTCTCGTCTGCTCTTG |
| *GSTU7* (At2g29420) | GTCGGAGGATTGAGATTG | ACAACTTTCATCGCCGTCAC |
| *GSTU13* (At1g27130) | GCCCTCCGTTCCTTCAATCC | CCTCCAAAGAACCCTAATCC |
| *GSTU19* (At1g78380) | GAACCCTATCCTTCCTTCTG | CTTGTTCCTCACCCTTTGTC |
| *GSTU22* (At1g78340) | AGACAAGAGCCCTTTGCTTC | GCTGTCTCTTGTTCCTCTC |
| *GSTU25* (At1g17180) | TCGACGAAGTTTGGCCTAGC | AACCTCGCTGAAGCATACAC |
| *GSTU27* (At3g43800) | GAAGAAGTGGTGGTGTTG | CCTCTTTGTCCACGTTCTTC |
| *GSTU28* (At1g53680) | CAAGGTTCTGGGCTGATTAC | CGAGTGCGTAGAACCAACTG |
| *DHAR2* (At1g75270) | TTCCCTACAAGACCCATCTG | TCAGCCACCCATTTGCCATC |
| *GSTF6* (At1g02930) | AAGCTTGGTGGCGCCGTTTG | ATGTCCTTGCCAGTTGAGAG |
| *GSTF7* (At1g02920) | CAAGGACATTGCGGGCATAG | TTAGGGCAATGAGGTCATCGCC |
| *GSTF8* (At2g47730) | GCGTCCTCGCTACTCTTTAC | GGAATCACCAGCCAAGAAC |
| *GSTF11* (At3g03190) | TCTTCGTCAGCCGTTTGGTC | GAGCCACAGCGTAGAAATAG |
| *GSTZ1* (At2g02390) | TGGTGGATGGAGATGTTGTG | TAACCCAGGCAGTCTTCTC |
| *GSTT2* (At5g41240) | ATGCGGATAGGATGTCACAG | ACACTTGCGTATGCTGAGG |
| *GSH1* (At4g23100) | GGAGATGAGAGGTGCTGATG | CCATCCTTTGCGAGTTTCAG |
| *GSH2* (At5g27380) | GATGCTCTCGTTTGGTCTTC | GGCTAACAAGACGACTAAGG |
| *ATG2* (At3g19190) | GGACGCCTGGATTGGATAGA | CGCTAGATGAGCTGGAGTTG |
| *ATG3* (At5g61500) | TACGATCTCGGCGTTCAAGG | GAAGCAGCTCTCCGTAGACA |
| *ATG5* (At5g17290) | GAAGAGAATGGCGAAGGAAG | GGCATCAAGATCACCGTTCA |
| *ATG6* (At3g61710) | TACCATCGTCGGCGTCGATT | GGCTGTTCAACCTGTGTCTG |
| *ATG8B* (At4g04620) | TGGCTGAGTCTACTCGTATC | CAGACATCAATGCCGCAGTT |
| *ATG8C* (At1g62040) | GGAAACGAGAAGCCCTAATC | GACATCATTGCAGCAGTTGG |
| *ATG8E* (At2g45170) | GAAGTACCTTGTGCCATCAG | ACGCTTGACATTAGCTCTCC |
| *ATG8F* (At4g16520) | TGAGAAGGCTGAGAAGAGTG | CGCTGTAAGTGACATAGAGG |
| *ATG8G* (At3g60640) | TGACAGAGTCCCGGTGATTG | ACCGATTGGTTGTGCCTAC |
| *ATG8H* (At3g06420) | GGTCCCACGAGACATGACTG | AACCCGTCTTCTTCCTTG |
| *ATG9* (At2g31260) | TGTTCTGGTATGCCGCTGTT | TTCTCTGCTCACGACGAGTT |
| *ATG12A* (At1g54210) | TCTCGCCAAACCCTGATG | CAAAGAGCAAGGCACAGATG |
| *ATG12B* (At3g13970) | GGTGGTGCTCCGATACTGAA | GTGAAGCTGCCGTCTTAGAA |
| *ATG18B* (At4g30510) | GCTGCTACTTAGCTGTTCCA | CCTAACCACGGCATAACTTC |
| *ATG18C* (At2g40810) | TGGTGGTGGACCTAACTCTC | ACTTGATACCGCCAGCCACT |
